# A 75-Year-Old Female with Dyspnea: An Unusual Presentation of Diffuse Large B-Cell Lymphoma

**DOI:** 10.7759/cureus.801

**Published:** 2016-09-22

**Authors:** Syed Daniyal Asad, Osama Mukarram, Khalid Abusaada

**Affiliations:** 1 Internal Medicine, Lahore Medical and Dental College, Lahore, Pakistan; 2 Internal Medicine, Texas Tech University Health Sciences Center; 3 Graduate Medical Education, Florida Hospital-Orlando

**Keywords:** diffuse large b cell lymphoma, non-hodgkin’s lymphoma, dyspnea, pericardial effusion, tuberculosis

## Abstract

Diffuse large B-cell lymphoma (DLBCL) is a common variant of non-Hodgkin lymphoma (NHL). It usually presents as a rapidly enlarging mass. Numerous presentations involving the gastrointestinal tract, bone, and the central nervous system have been reported in the past including both primary and secondary involvement of organs.

A 75-year-old lady was found to have a pericardial effusion while being evaluated for shortness of breath. A therapeutic pericardial tap was positive for pan-B cell markers. The patient's detailed radiological studies failed to show a primary tumor. We report this unusual presentation of DLBCL as a pericardial effusion without a primary source in a patient with mild shortness of breath.

## Introduction

Diffuse large B-cell lymphoma (DLBCL) is a histological subtype of non-Hodgkin’s lymphoma (NHL). It is a neoplasm of largely transformed B cells and represents approximately one-third of all the lymphoma cases. Fifty-five percent of the individuals affected are male. The patients typically present with a rapidly enlarging symptomatic mass. Up to one-third of the patients also have B symptoms (fever, weight loss, and night sweats) at the time of presentation [1].

DLBCL usually involves the lymph nodes; however, almost half of the cases are extranodal. The common sites include the gastrointestinal tract, bone, and central nervous system (CNS). Approximately 60% of the patients present with advanced stage DLBCL (Stage III or IV disease) [2]. We report an interesting and unique presentation of DLBCL as a pericardial effusion with no obvious source. Informed consent was obtained from the patient for this study.

## Case presentation

A 75-year-old female was referred to a medicine clinic in Pakistan for medical clearance for a planned cholecystectomy. She had symptomatic cholelithiasis. The patient also complained of dyspnea. She had been feeling increasingly short of breath for the past two months without any functional impairment. She did not report having any B symptoms. Her past medical and surgical history was not significant for hypertension, diabetes, ischemic heart disease, or malignancies. The patient's blood pressure and heart rate were in the normal range. The physical examination was unremarkable except for mild tenderness in the right upper quadrant of the abdomen, which raised suspicion for acute cholecystitis.

**Figure 1:**
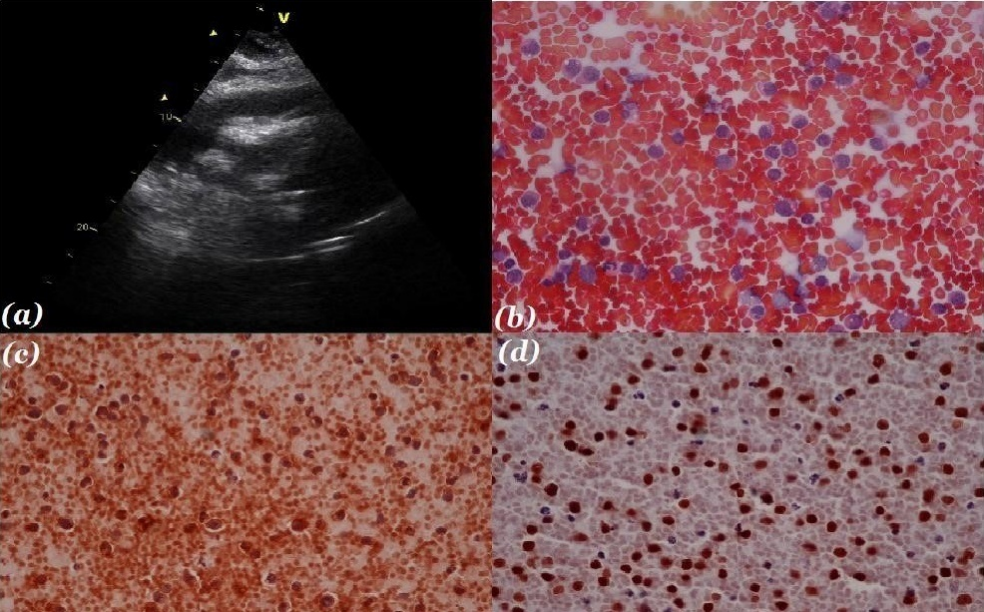
Analysis of the pericardial effusion Figure 1 (a) Echocardiogram showing pericardial effusion. (b) Pap stain of the pericardial effusion showing malignant lymphoma cells. (c) Immunohistochemical analysis of the pericardial effusion showing CD20+ cells. (d) Immunohistochemical analysis of the pericardial effusion showing Pax-5 cells.

The patient's complete blood count and blood chemistries were within normal limits. However, a raised lactate dehydrogenase (LDH) (1129 mg/dl) was noted. An electrocardiogram (ECG) was obtained, and it showed low voltage regular QRS complexes in the limb and precordial leads. The chest X-ray demonstrated an enlarged cardiac silhouette. In addition to findings consistent with cholelithiasis, her abdominal ultrasound also revealed trace right-sided pleural effusion, moderate pericardial effusion, and minimal ascites. As a part of further workup, an ECG (Figure 1) was done which revealed a moderate pericardial effusion with no evidence of tamponade and an ejection fraction of 55%. Based on this, therapeutic pericardiocentesis was performed and 800 mL of blood-tinged pericardial fluid was aspirated. The fluid was sent for microbiological and cytological evaluation. The post-procedure ECG demonstrated an improved ejection fraction with minimal residual effusion. The results of the effusion smear and culture were negative for the acid-fast bacilli (AFB). However, the cytological findings were strongly suspicious for a lymphoproliferative neoplasm (Figure 1).

The immunohistochemical analysis of the pericardial fluid (Figure 1) showed the infiltrate to be strongly positive for pan-B cell markers (CD20 and Pax-5) and leukocyte common antigen (LCA). It was negative for epithelial markers (AE1/AE3) and blast cell markers (CD34 and myeloperoxidase (MPO)). Therefore, a final diagnosis of aggressive B-cell NHL was made. 

In light of the cytological and immunohistochemical evidence, the patient was re-examined thoroughly and a full body computerized tomography (CT) scan was done to search for a primary mass lesion. The CT scan demonstrated bilateral minimal pleural effusion with a mild pericardial effusion. The spleen was unremarkable and there was an absence of significant lymphadenopathy or abnormal mass lesions anywhere in the body.

The oncology department was consulted, and it tentatively staged the disease as Stage IE but wanted to perform additional tests including a bone marrow biopsy. The patient was given the first dose of chemotherapy with rituximab, vincristine, and prednisone since the patient refused the standard R-CHOP regimen (rituximab with cyclophosphamide, doxorubicin, vincristine, and prednisone) citing financial concerns. She was offered help in arranging financial assistance by the Patient Welfare Department of the hospital. However, she wanted to be discharged after receiving the first dose of chemotherapy. The patient was counselled on the importance of treatment but she decided to leave against medical advice. It was planned to obtain a bone marrow aspirate at the next follow-up visit but the patient did not maintain follow-up, though she was advised to follow-up.

## Discussion

In South Asia, an incidental finding of pericardial effusion is commonly associated with tuberculosis. Tuberculous effusion was in the differential diagnosis along with primary cardiac lymphoma (PCL), primary effusion lymphoma (PEL), and malignant pericardial effusion. A negative AFB smear and culture were used to rule out the possibility of tuberculosis.

PCL most often presents as a mass in the atria or ventricles. In a review of 48 cases of PCL in immunocompetent patients by Ceresoli, et al., more than 80% arose in the right heart chambers as a mass with or without pericardial effusion [3]. Primary cardiac DLBCL has been documented as an atrial mass in more than 90 cases, the most recent by Johri, et al. [4] in 2009. Interestingly, no such mass was visible on the CT scan or echocardiogram of our patient. Johri, et al. also pointed out that it is most often found in immunocompromised individuals [4]. Our patient's physical examination and laboratory data did not show any signs of immunosuppression. However, an endomyocardial biopsy would have been ideal to make this diagnosis, as primary cardiac DLBCL can present with an unexplained pericardial effusion. DLBCL presenting with a pericardial effusion has been reported by Yap LB, et al. [5].

PEL was considered as the effusion involved multiple serous spaces. However, PEL is typically negative for CD20 and is almost exclusively seen in human immunodeficiency virus (HIV) positive patients [6]. Our patient’s HIV status could not be determined but the presence of CD20 marker was considered sufficient evidence to rule out PEL. The tumor cells in DLBCL generally express pan-B cell antigens (CD19, CD20, CD22, CD79a, Pax-5), as well as CD45 [7]. 

Various other presentations of DLBCL have been reported. A primary bone marrow presentation of DLBCL has been noted by Roth, et al. [8]. They reported 13 cases involving the bone marrow out of which 12 presented with cytopenia. The simultaneous detection of multiple extranodal involvements at presentation is uncommon, but Kim, et al. [9] have described a case of DLBCL presenting with simultaneous bone and stomach involvement.

Ideally, the patient should have received multiple cycles of the standard R-CHOP regimen (Rituximab with cyclophosphamide, doxorubicin, vincristine, and prednisone) as it has consistently shown to improve five-year survival in elderly patients with DLBCL [10]. A positron emission tomography (PET) CT scan would have also helped in the evaluation. However, the hospital lacked a PET-CT scan facility. In the developing world, a dearth of treatment and diagnostic resources often forces physicians to come up with management plans that may not be ideal but are tailored to provide maximum benefit to patients.

## Conclusions

In the authors’ opinion, although DLBCL is a rare cause of pericardial effusion, it still needs to be considered in the differential diagnosis of pericardial effusion of unclear etiology, especially in tuberculosis-prevalent areas, as the clinical presentation often overlaps.
